# *Arabidopsis thaliana* dehydroascorbate reductase 2: Conformational flexibility during catalysis

**DOI:** 10.1038/srep42494

**Published:** 2017-02-14

**Authors:** Nandita Bodra, David Young, Leonardo Astolfi Rosado, Anna Pallo, Khadija Wahni, Frank De Proft, Jingjing Huang, Frank Van Breusegem, Joris Messens

**Affiliations:** 1 Center for Structural Biology, VIB, 1050 Brussels, Belgium; 2 Brussels Center for Redox Biology, 1050 Brussels, Belgium; 3 Structural Biology Brussels, Vrije Universiteit Brussel, 1050 Brussels, Belgium; 4 Center for Plant Systems Biology, VIB, 9052 Gent, Belgium; 5 Department of Plant Biotechnology and Bioinformatics, Ghent University, 9052 Gent, Belgium; 6 Research Group of General Chemistry, Vrije Universiteit Brussel, 1050 Brussels, Belgium

## Abstract

Dehydroascorbate reductase (DHAR) catalyzes the glutathione (GSH)-dependent reduction of dehydroascorbate and plays a direct role in regenerating ascorbic acid, an essential plant antioxidant vital for defense against oxidative stress. DHAR enzymes bear close structural homology to the glutathione transferase (GST) superfamily of enzymes and contain the same active site motif, but most GSTs do not exhibit DHAR activity. The presence of a cysteine at the active site is essential for the catalytic functioning of DHAR, as mutation of this cysteine abolishes the activity. Here we present the crystal structure of DHAR2 from *Arabidopsis thaliana* with GSH bound to the catalytic cysteine. This structure reveals localized conformational differences around the active site which distinguishes the GSH-bound DHAR2 structure from that of DHAR1. We also unraveled the enzymatic step in which DHAR releases oxidized glutathione (GSSG). To consolidate our structural and kinetic findings, we investigated potential conformational flexibility in DHAR2 by normal mode analysis and found that subdomain mobility could be linked to GSH binding or GSSG release.

Oxidative stress has a significant impact on the cellular environment of organisms. Control of the reactive oxygen species (ROS) that cause such stress is essential for effective redox homeostasis. Generation of ROS can occur endogenously through leakage from respiratory complexes or photosystems, or can be induced by external stressors, such as UV radiation, drought, temperature extremes, or elevated salinity^1^,^2^,^3^,^4^,^5^. Once released, ROS inflict cellular damage through oxidative inactivation of enzymes, metal oxidation, and mutagenesis^6^,^7^. Soluble small-molecule antioxidants, such as ascorbate (AsA) or glutathione (GSH), neutralize ROS either by direct reduction or by acting as cofactors for redox enzymes, such as peroxidases^8^,^9^,^10^. Cellular compartments maintain a reducing environment by constant recycling of oxidized antioxidants back to their reduced forms, a reaction catalyzed by glutathione reductase (GR) in the case of oxidized glutathione (GSSG) and dehydroascorbate reductase (DHAR) for dehydroascorbate (DHA), the oxidized form of AsA^11^. The intracellular concentration of GSH and AsA in plants are typically maintained within the range of 2–6 mM and 2–25 mM, respectively. GSH (5 mM) is able to directly reduce DHA through a non-enzymatic mechanism, albeit at a rate of 17 nmol min^−1^ 12,13, which is significantly lower than the reduction catalyzed by DHAR (20–370 μmol min^−1^ mg^−1^)^14^.

AsA typically behaves as a single-electron donor and is converted to its semi-oxidized radical form, monodehydroascorbate (MDHA) upon ROS reduction. Two molecules of MDHA then disproportionate into AsA and DHA or, alternatively, MDHA can be enzymatically reduced to AsA by MDHA reductase^15^. Whereas GSH is relatively stable in its oxidized form, DHA undergoes irreversible hydrolysis to diketogluonate (DKG)^16^, and therefore, rapid reduction of DHA in cells is critical for effective AsA recycling. AsA is the major antioxidant of plants and, accordingly, the majority of the characterized DHAR enzymes are of plant origin. Plant DHAR enzymes contain a conserved catalytic motif CPFS/C and are largely categorized into four isoforms, DHAR1, DHAR2, DHAR3 and DHAR4^17^. To date, four independent structures of plant DHAR have been deposited in the Protein Data Bank: the crystallographic structures of *Oryza sativa* (rice) (OsDHAR1; PDB, 5D9T)^18^, *Pennisetum glaucum* (pearl millet) (PgDHAR1; PDB, 5EV0, 5IQY), the nuclear magnetic resonance solution structure of DHAR3A from *Populus trichocarpa* (black cottonwood) (PtDHAR3A; PDB, 2N5F)^19^, and the recently deposited crystal structure of *Arabidopsis thaliana* DHAR1 (AtDHAR1; PDB, 5EL8)^20^. In addition, crystal structures of *P. trichocarpa* GST Lambda (PtGSTL)^21^ and *Homo sapiens* GST Omega (HsGSTO)^22^ with DHAR activity have been determined with GSH bound at the catalytic cysteine. As the AtDHAR1 structure is yet to be published, we will not discuss it here.

DHAR is also structurally homologous to chloride intracellular channel (CLIC) proteins which, in their soluble globular state, have been shown to exhibit low levels of DHAR activity, although they primarily function as multimeric membrane-integrated ion channels^23^,^24^,^25^. Intriguingly, AtDHAR1 also appears to be capable of transmembrane ion conductance, but the relevance of such activity has still to be explored^26^.

Recently, a mechanism for DHA reduction by DHAR has been proposed based on the oxidized and AsA-bound structures of OsDHAR1^18^. Here, from the structural and biochemical investigation of *Arabidopsis* DHAR2 (AtDHAR2), we provide further support for this mechanism and use elastic network modeling to explore the apparently allosteric behavior in the enzymatic DHAR2 mechanism.

## Results and Discussion

### The kinetic parameters and the release of GSSG as reaction product

**Figure 1:**
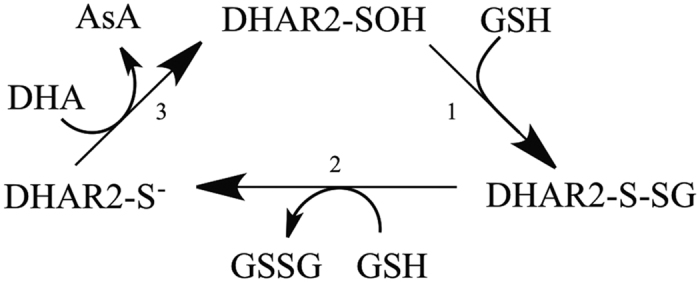
Reaction scheme for the catalytic cycle of DHA reduction by DHAR2. In a mechanistic scheme, the ping-pong mechanism for the enzymatic reduction of DHA is shown. DHAR2 is sulfenylated at the catalytic cysteine (Cys20) and GSH performs a nucleophilic attack on the sulfenylated Cys20 to form a mixed disulfide, DHAR2-S-SG (step 1). A second GSH molecule reacts with the mixed disulfide, producing GSSG and the cysteine is released in its reduced thiolate form (step 2). DHA enters the active site of the reduced form of DHAR2 and is converted to AsA (step 3).

Previously, DHAR has been reported to have a bi-uni-uni-uni ping-pong enzymatic mechanism, with GSH and DHA interacting with the catalytic cysteine (Cys20 in AtDHAR2) in separate, sequential binding events (Fig. 1)^27^. This catalytic cysteine is essential for enzymatic activity, and mutation to a serine (to mimic the catalytic motif common to GSTs) has been shown to abolish the DHA reductase activity^27^. The reduction of DHA by DHAR has been proposed to result in the formation of a sulfenic acid at the catalytic cysteine, based on the crystallographic identification of Cys20 over-oxidation in OsDHAR1 upon soaking crystals with DHA^17^. A sulfenic acid at Cys20 of AtDHAR2 has also been identified in *Arabidopsis* cell suspensions subjected to oxidative stress^28^. Cysteinyl sulfenic acids readily form mixed disulfides with GSH under physiological conditions, thereby protecting against irreversible over-oxidation of the cysteine sulfur^29^,^30^,^31^,^32^. Such S-glutathionylation of a sulfenylated Cys20 comprises reaction step 1 of the mechanistic scheme (Fig. 1), of which the formation in AtDHAR2 had previously been confirmed by mass spectrometric analysis^28^. Nucleophilic attack of a second molecule of GSH on the Cys20 mixed disulfide then generates the reduced enzyme form with a thiol at catalytic Cys20 (Fig. 1, step 2) and releases GSSG as by-product. However, as of yet, no direct biochemical evidence exists to support this mechanistic step.

**Figure 2:**
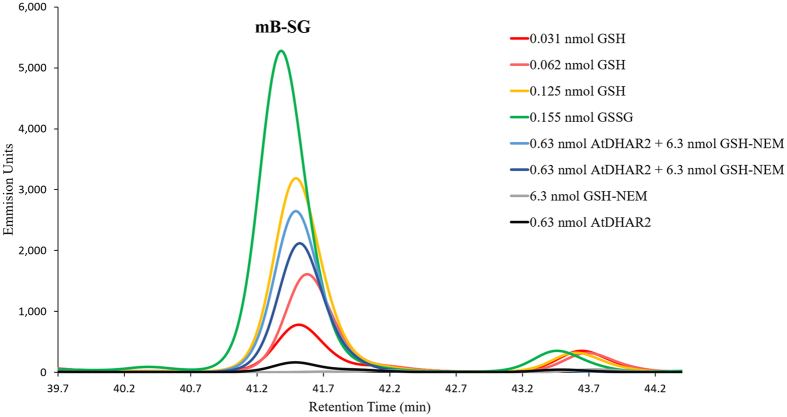
Reversed-phase fluorescence elution profile of the glutathione-monobromobimane (GS-mBBr) derivatives. Fluorescence peaks were assigned according to known standards. GSH was alkylated prior to reduction of GSSG, therefore, no mBBr derivatization of the GSH substrate was observed, as shown by the negative control of 6.3 nmol GSH-N-ethylmaleamide (NEM) (grey trace). AtDHAR2-SG without addition of free GSH was used as a negative control (black trace). Concentrations are given as final molar values for the total injected sample. A full elution trace is available in the Supporting information (Fig. S6).

To experimentally validate reaction step 2, we detected the generation of GSSG from S-glutathionylated AtDHAR2 through thiol-labeling of the GSSG reduction product, GSH, by monobromobimane (mBBr). This mBBr is an essentially non-fluorescent compound that can form a conjugate with GSH via nucleophilic attack of the glutathione thiolate at the alkyl halide with formation of a thioether bond. The resulting conjugate is highly fluorescent and mBBr derivatization has been effectively applied in the quantification of free GSH in the pmol range^33^. The reactivity of mBBr is largely thiol specific, and conjugations to amines or other nucleophiles occur at a markedly low rate. Analysis of the mBBr conjugates by reversed-phase high-performance liquid chromatography (RP-HPLC) allows the separation and distinction of hydrolysis products and other contaminants from the fluorescent mBBr-glutathione conjugate (mB-SG). By treating pre-glutathionylated AtDHAR2 with excess GSH and then blocking free thiols with an *N*-ethylmaleamide (NEM), the formation of GSSG was detected by reduction and subsequent mBBr derivatization (Fig. 2). From this, it could be concluded that GSH reacts with the mixed-disulfide of S-glutathionylated DHAR2, producing GSSG and a reduced thiol at Cys20.

**Figure 3:**
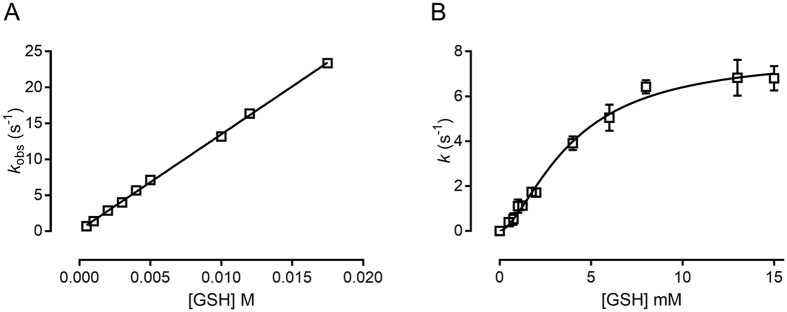
Pre-steady-state and steady-state kinetics of AtDHAR2. **(A)** Stopped-flow analysis of the reaction of GSH with AtDHAR2:GSH. A linear dependence of the observable rate constants allowed the calculation of a second-order rate constant of 1331 ± 13 M^−1^ s^−1^ for the conversion of S-glutathionylated DHAR2 to its reduced form. **(B)** A sigmoidal rate variance with respect to GSH concentrations with a fixed saturated concentration of DHA (200 μM), represented as a rate constant *k* (s^−1^) versus the GSH concentration in mM.

With the understanding that GSSG can be generated from S-glutathionylated AtDHAR2, we determined the catalytic rate of reaction step 2 by exploiting the intrinsic fluorescence of AtDHAR2 (which contains four tryptophan residues) in a stopped-flow analysis of the GSSG formation from a AtDHAR2-SG mixed-disulfide (Fig. 3A). A second-order rate constant of 1331 ± 13 M^−1^ s^−1^ was found and a rate of 5.6 (±0.02) s^−1^ at 4 mM GSH, which is an assumed average physiological concentration of GSH in the *Arabidopsis* cell cytoplasm^12^.

Characterization of the steady-state kinetics of AtDHAR2 under saturated DHA conditions (200 µM with *K*_0.5_ = 23 ± 1 μM for DHA)^28^ with variable concentrations of GSH revealed a sigmoidal behavior of velocity dependence, indicative of cooperative interplay (Fig. 3B). Fitting data to equation 1 (see Materials and Methods) yielded a *K*_0.5_ = 3.9 ± 0.4 mM, V_max_ = 20 ± 1 μmol min^−1^ mg^−1^, *k*_cat_ = 7.8 ± 0.4 s^−1^, and h = 1.6 ± 0.15. The Hill coefficient indicates either a positive heterotropic effect of GSH on DHA binding, or a positive homotropic effect of GSH on the affinity of the second molecule of GSH. Previous kinetic characterization of AtDHAR2 revealed a Hill coefficient of 2.6 when varying the concentration of DHA^28^. Taken together, AtDHAR2 is the first DHAR to display allosteric behavior. The V_max_ of AtDHAR2 is markedly lower than that reported for OsDHAR1 (350 μmol min^−1^ mg^−1^), although the kinetic parameters of OsDHAR1 were measured at pH 8 (the catalytic optimum)^34^, whereas the steady-state kinetics of AtDHAR2 were measured at pH 7 (Table S1). The catalytic activity of OsDHAR1 was almost 50% lower than the full activity at pH 7 and, therefore, caution should be taken when directly cross-comparing catalytic parameters of DHARs^35^. The *k*_cat_/*K*_M_ of OsDHAR1 for DHA is within the same range as the *k*_cat_/*K*_M_ for GSH, 3.91 × 10^5^ M  s^−1^ and 1.37 × 10^5^ M^−1^ s^−1^, respectively, showing a comparable substrate specificity^34^. In contrast, the *k*_cat_/*K*_0.5_ of AtDHAR2 for DHA was significantly higher than for GSH, 3.39 × 10^5^ M^−1^ s^−1^ and 2 × 10^3^ M^−1^ s^−1^, respectively, hinting at a considerably higher substrate specificity for DHA in the case of AtDHAR2.

The *k* of reaction step 2, i.e., AtDHAR2 reduction and GSSG formation at 4 mM GSH (as derived from the stopped-flow analysis), is relatively close to the steady-state *k* value at an equivalent GSH concentration (5.6 ± 0.02 s^−1^ and 3.8 ± 0.2 s^−1^, respectively), and at GSH concentrations below 4 mM, k values from stopped-flow and steady-state converge even further. This indicates that at sub-physiological GSH concentrations (below 4 mM), reaction step 2 is probably the rate-limiting step in the catalytic cycle of AtDHAR2. Allosteric enzymes in a ping-pong mechanism are characterized by stable interconvertible macro-states throughout the reaction sequence, usually related to chemical modifications of the original enzyme, such as enzyme reduction^36^. As stopped-flow analysis measures the overall change in protein fluorescence, and considering the kinetic allostery implied in the steady-state kinetics, it is possible to conclude that the rate limitation is related to structural changes.

### Overall structure of AtDHAR2

**Figure 4:**
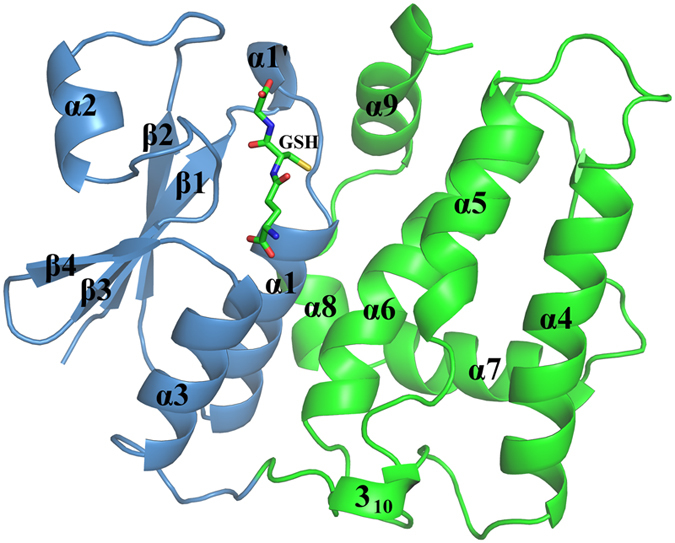
Crystal structure of the GSH-bound AtDHAR2. The N-terminal thioredoxin-like domain (blue) and the C-terminal helical domain (green) are shown.

The crystal structure of AtDHAR2 in complex with GSH was solved by molecular replacement to a resolution of 2.3 Å in space group *P*2_1_22_1_. Due to the inherent flexibility of the terminal regions of the polypeptide chain, residues 1–4 and 212–213 of the 213-residue protein were undefined by electron density. AtDHAR2 crystallized as a monomer and exhibited the classic structural architecture common to the GST super-family, with an all-helical C-terminal domain, and a thioredoxin-like N-terminal domain consisting of a mixture of β-sheets and α-helices (Fig. 4). The overall structural fold of AtDHAR2 is almost identical to that of the structures of OsDHAR1 and PgDHAR1, albeit with the inclusion of an additional short-chain helical turn preceding the α1-helix (Fig. 4). The AtDHAR2 structure superposes well with those of OsDHAR1 and PgDHAR1, aligning with a root-mean-square deviation of 0.85 Å over 193 C^α^ and 0.8 Å over 191 C^α^, respectively, with structural differences arising primarily across the α2-helix-containing region. Structural overlay plant DHARs, plant GSTL1, human GSTO2, and human CLIC1 revealed a localized conformational variability in the α2-helical region (Fig. S1). Comparative analysis of the structures of AtDHAR2 and OsDHAR1 by the DynDom server (http://fizz.cmp.uea.ac.uk/dyndom/)^37^ identified the α2-helix to be a hinged subdomain, spanning residues 32–63.

### Interactions of a putative hinged subdomain with G-site GSH

Glutaredoxins (Grx) and the GST enzyme family bind GSH in the solvent-exposed active site cleft at a region designated the G-site. Residues involved in the binding of GSH at the G-site are all located within the N-terminal thioredoxin-like domain, and a core interaction invariably occurs between the thiol of GSH and the catalytic Cys/Ser/Tyr of the α1-helix.

**Figure 5:**
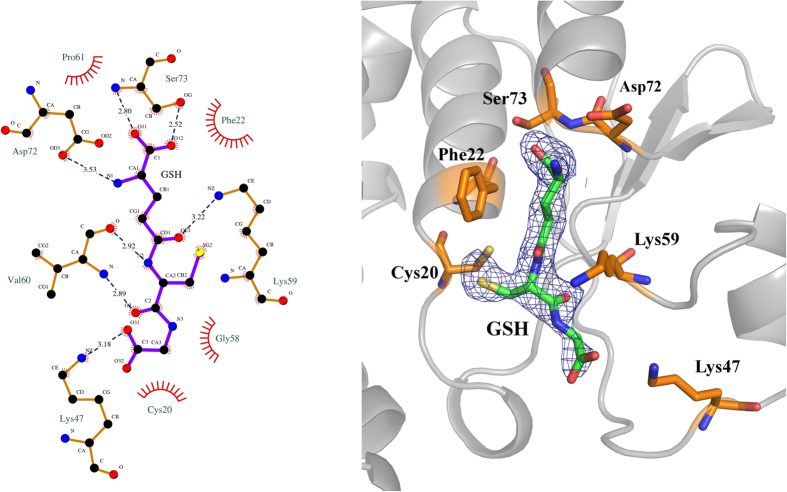
Schematic representation of the G-site GSH environment in AtDHAR2. The bonding environment of GSH at the G-site of AtDHAR2 is displayed in LIGPLOT (left panel) and PyMOL (right panel)^36^. H-bonding and salt bridge interactions are illustrated in LIGPLOT by blue dashed lines. The *mF*_*O*_*-DF*_*C*_ omit map for GSH is defined by a blue mesh contoured at 3σ. Waters interacting with GSH are omitted.

GSH was placed into the available electron density of the *mF*_*O*_*-DF*_*C*_ difference map at the G-site of AtDHAR2 at full occupancy in a non-covalently bound state. The cysteinyl sulfur of GSH was in close proximity (2.8 Å) to the sulfur atom of Cys20, thereby indicating that the GSH was likely engaged in a mixed disulfide (for which a maximum bond length of 2.3 Å is commonly applied) with Cys20 before disulfide cleavage by X-ray irradiation, a well-characterized phenomenon^38^,^39^,^40^. As an indication of the relative stability of GSH binding, the average B-factor for the G-site GSH (34.9 Å^2^) is comparable to that determined for the polypeptide main-chain atoms (34.8 Å^2^). The GSH γ-glutamyl is particularly well stabilized, as evidenced by low B-factor values of 24–29 Å^2^, accepts H-bonds to its carboxylate from a water molecule and from the backbone amide and side-chain hydroxyl of Ser73, and forms a salt bridge interaction with Asp72 via its amine group (Fig. 5). The γ-glutamyl group is also engaged in van der Waals interactions with Phe22, and hydrogen-bonds the side-chain amine of Lys59 by its γ-glutamyl peptide carbonyl. The central cysteinyl region of the GSH molecule is stabilized by hydrogen bonding of the carbonyl and amide of the cysteine with the backbone amide and carbonyl groups of Val60. The glycinyl group of GSH is less tightly bound, as reflected by elevated B-factors of 39–48 Å^2^, forming a single salt bridge with the side-chain amine of Lys47 through its carboxylate. Among the GST enzyme family, Asp72 and Ser73 are well conserved and are also present in the GSH-bound structures of PtGSTL and HsGSTO2, whereas Lys47 and Lys59 are less well conserved, although they are often substituted by equivalently charged residues. In the case of HsGSTO2, Lys47 is conserved (Lys59 in HsGSTO2) and forms a salt bridge interaction with GSH, and a histidine residue occupies the Lys59 position, but without forming an interaction with G-site GSH^22^. Similarly, in the PtGSTL structure, an arginine substitutes the salt bridge by Lys47, and although Lys59 is conserved (Lys78 in PtGSTL1), it does not form a hydrogen bond with GSH^21^. In the S-glutathionylated structure of HsCLIC1 (PDB, 1K0N), there are surprisingly few interactions with GSH. H-bonds are formed only through Thr77 (CLIC1 numbering) and the peptide backbone carbonyl of Leu54 (CLIC1 numbering), with Lys49 (Lys47 in AtDHAR2) too distant for salt bridge formation and Lys59 is not conserved (Glu63 in CLIC1)^41^.

### The H-site and the nature of DHA binding

In addition to the G-site, the GST enzyme family also possesses a second substrate binding site, designated the H-site, which mostly consists of residues from the C-terminal domain. In contrast to the conserved nature of the G-site, the H-site typically exhibits more structural plasticity among GSTs, relating to a substrate specificity variation. Based on the crystal structure of OsDHAR1, both DHA and a secondary GSH molecule are able to bind at the H-site of DHAR, albeit not simultaneously. From the structure of the AsA-bound OsDHAR1, Lys8, Asp19, and Lys210 are identified as forming H-bonds with AsA^17^. The structure of PgDHAR1 also supports the role of Lys8 and Asp19 in DHA binding, with glycerol as a mimic of the 1,2-dihydroxyethyl arm of AsA^20^. In addition to the charged residues mentioned above, hydrophobic van der Waals interactions from Pro21, Phe104, and Trp207 contribute to the binding of AsA in the crystal structure of the AsA-bound OsDHAR1^18^. All the residues observed to interact with AsA in the OsDHAR1 structure are conserved among DHARs (Fig. S1).

Comparison of the H-site region of AtDHAR2 to that of PgDHAR1, OsDHAR1, and PtDHAR3A reveals a distinctive difference in the conformation of Asp19. The introduction of the α1’-helix in AtDHAR2 (Fig. 1) alters the peptide torsion of Asp19 in such a way as to re-direct its side-chain carboxylate toward the protein interior, forming a hydrogen bond with His160 (Fig. S2). In the case of OsDHAR1, PgDHAR1, and PtDHAR3A, the side-chain of Asp19 is orientated outward into the H-site pocket and a polar interaction between His160 and Asp19 is instead formed via the backbone carbonyl of Asp19^19^ (Fig. S3). As Asp19 is proposed to be a significant residue in the binding and stabilization of DHA for catalytic reduction^18^,^20^, preclusion of its carboxylate side-chain from the H-site-binding region in AtDHAR2 complicates understanding of its role in DHA reduction.

In the proposed molecular mechanism of enzymatic DHA reduction^18^, Lys8 has a central function in both binding and protonation of DHA and its site-directed mutagenesis in both OsDHAR1 and *Populus tomentosa* DHAR2 has been shown to significantly reduce the catalytic efficiency^18^,^42^. However, although Lys8 is conserved in AtDHAR2, it is instead positioned at the beginning of the β1-strand, distal from the active site cleft and 19 Å C^α^-C^α^ from the positioning of Lys8 of OsDHAR1, and the equivalent position occupied by a glycine.

It is notable that in PtGSTL1, which also displays DHAR activity, Asp19 or Lys8 are not conserved, and are instead substituted by a threonine and serine, respectively. With the assumed functional significance of Lys8 and Asp19, the preclusion of such residues from the active site environment of AtDHAR2 would be expected to reduce its catalytic efficiency with respect to DHA. However, the kinetic parameters defined for AtDHAR2 reveal a markedly enhanced substrate specificity toward DHA relative to GSH, whereas such a difference in substrate specificity is not evident for OsDHAR1.

### Normal mode analysis to assess structural flexibility

The allosteric behavior of DHAR2 activity could be ascribed to a structural rearrangement during GSH binding and/or GSSG release. The most likely place for such a structural rearrangement to occur would be the α2-helix and its connecting loops, in which many of the residues involved in G-site GSH-binding are located. The inherent flexibility of the α2-helix in *Homo sapiens* GST Pi (HsGSTP1–1) has been previously highlighted on the basis of high crystallographic temperature factors^43^. For PgDHAR1, OsDHAR1, and AtDHAR2, crystallographic temperature factors for the α2-helix are within the range of the structural average, however, this is because intermolecular crystal contacts formed at the α2-helix interface provide stabilizing interactions and decrease the domain flexibility, thereby reducing the respective temperature factors.

Computational simulation of macromolecular flexibility can provide valuable insight into the range and direction of potential motions, and one such computational approach is normal mode analysis (NMA). NMA uses a coarse-grained structural model, in which residues are considered as nodes of equal mass and a single-parameter potential energy function describes interactions between nodes within a defined interaction distance. Collective molecular motions are defined through eigenvectors across the potential energy matrix, corresponding to the vibrational normal modes, and through eigenvalues, which are the associated frequencies. Despite the significant simplification of energetic parameters and lack of explicit solvent, the lowest frequency modes calculated by NMA have been found to be particularly effective in predicting large-scale macromolecular motions^44^.

Two separate NMA web servers were used to assess the structural dynamics of AtDHAR2; elNémo (http://igs-server.cnrs-mrs.fr/elnemo/index.html)^45^ and ANM 2.0 (http://anm.csb.pitt.edu)^46^. The two lowest-frequency normal modes calculated described either a twisting motion or breathing motion around an axis along the groove between the N-terminal thioredoxin-like domain and the all-helical C-terminal domain, relating to a partial opening and closing of the active site cleft. The largest amplitude of atomic displacement was observed in the α2-helix region and in a solvent-exposed loop connecting the α4- and α5-helices (Fig. S4). As this loop does not contain residues involved in substrate binding, it was not considered to be a functionally significant region. These findings correlate well with the more in-depth molecular dynamics simulations used previously in the study of CLIC1, in which the same two types of concerted motions were identified and significant structural change of the α2-helix were observed as well^47^. Molecular dynamics simulations performed on HsGSTP1-1 have also found the largest structural change in the α2-helix^48^.

### Motion of the α2-helix during the catalytic mechanism

Allosteric behavior in monomeric enzymes is relatively rare, and, in the context of ping-pong kinetics, interpretation of such allosteric behavior is largely a speculative task. One possible explanation for the allosteric behavior of AtDHAR2 takes GSH as a positive regulator, in which the G-site S-glutathionylation increases the GSH affinity for the H-site. Alternatively, the allosteric mechanism of AtDHAR2 may be purely related to structural changes, in which the α2-helix region dynamics regulate the affinity for GSH/GSSG. The tendency of the α2-helical region to switch conformations could be linked to the presence or absence of the G-site GSH, where H-bonding and salt bridge interactions between GSH and the α2-helical region would cause one conformation to be favored over the other. In the GSH-bound AtDHAR2, significant steric overlap (>0.5 Å) occurs between Val44 and Asp46 located within the α2 hinge region, indicative of a strained conformation. This observation could imply an induced-fit conformation in the S-glutathionylated state of the enzyme with a G-site-apo form representing an energetically favored resting conformation. Potential conformational flexibility of the α2-helix of GSTs has been studied in depth for HsGSTP1-1, with conclusions supporting an induced-fit model for GSH binding^48^,^49^,^50^,^51^. Interestingly, positive cooperativity in the catalytic activity of GSTP1-1 was shown to be induced upon site-directed mutation within the α2-helix, increasing both the *K*_0.5_^GSH^ and the Hill coefficient^52^. For the *Phanerochaete chrysosporium* GST-class enzyme, Ure2p, which exhibits DHAR activity, GSSG-bound and apo crystal structures show that the α2-helix becomes disordered in the absence of bound GSSG, indicating a more extreme flexibility in this region which depends on the binding of GSSG^53^. GSSG has been shown to be a competitive inhibitor of the DHAR activity with respect to DHA, with a reported 73% activity reduction at 2.5 mM GSSG^27^,^42^; therefore, rapid diffusion of GSSG from the active site could be an important factor and the α2-helix dynamics may release GSSG from the active site-binding cleft to allow DHA binding. Another possible functional implication of structural dynamics could be a cooperative relationship between the α2-helix and the α1′-helix. The α1′-helix is an unusual structural feature among the GST enzyme family. The formation of this coil precludes the side-chain Asp19 from interaction with H-site-bound DHA and also forces a repositioning of the conserved Lys8 to a location distal from the active site. With the inward conformation of the α2-helix in AtDHAR2, uncoiling of the α1′-helix is not possible –which would reorient the Asp19 side-chain into the active site– due to inevitable steric interference of the N-terminal loop of the α2 hinge region. If the α2-helix were to shift outward to a conformation more closely resembling that of OsDHAR1 or PgDHAR1, the steric hindrance would be relieved, allowing uncoiling of α1′.

Examples within the GST family of structural flexibility in the α2-helix provide a precedent for conformational change in the case of AtDHAR2, and prior evidence of an induced-fit mechanism and induced positive cooperativity in GSTP1-1 supports the possible influence of this helix on the allosteric behavior of AtDHAR2^49^,^52^. However, as GSTP1-1 functions as a homodimer, direct comparison to the monomeric DHAR should be viewed with caution. AtDHAR2 is the first monomeric GST-family enzyme shown to display an allosteric enzymatic behavior, though whether this allostery is common to other isoforms of DHAR or specific to AtDHAR2 alone remains an open question.

## Materials and Methods

### Cloning, purification, and glutathionylation of AtDHAR2

Recombinant AtDHAR2 was expressed in *Escherichia coli* C41 (DE3) and purified as described^28^. Glutathionylated AtDHAR2 was prepared by a 5-min pre-equilibration of 20 μM DHAR2 with 1 mM GSH, followed by a 30-min incubation with 1 mM H_2_O_2_ at room temperature. Excess GSH was removed by gel filtration with a 16/60 Superdex75 column (GE Healthcare) equilibrated in 20 mM Hepes (pH 7.5), 150 mM NaCl, and 1 mM EDTA. The eluted protein was concentrated by centrifugal filtration.

### Derivatization of GSH with monobromobimane (mBBr)

For mBBr derivatization of GSSG-derived GSH, enzyme samples, known standards, and controls were prepared as described previously^54^,^55^. Samples of 0.001–1 mM GSH, 1–100 μM GSSG, 100 μM of glutathionylated DHAR2, and 100 μM of glutathionylated DHAR2 with 1 mM GSH were prepared in 300 mM MOPS (pH 7), 150 mM NaCl, and 1 mM EDTA (freshly flushed with argon gas). Of each sample, 20 μL was mixed with 25 μL acetonitrile, 5 μL of 10 mM N-ethylmaleamide for alkylation of free thiols, and incubated at room temperature for 30 min. Sample pH was brought to pH 10.5 by 0.5 M NaOH and, after 5 min, the pH was readjusted to pH 7 with 1 M HCl. After centrifugation for 5 min at 20,000 × *g*, tris(2-carboxyethyl)phosphine was added to a final concentration of 1 mM and samples were incubated for 20 min. Subsequently, mBBr (in acetonitrile) was added to a final concentration of 10 mM and samples were incubated in the dark at room temperature for 20 min. The mBBr derivatization was stopped by addition of 40 mM methane sulfonic acid at a 10:1 acid:sample ratio. Protein aggregates were removed by centrifugation (5 min, 20,000 × *g*) and syringe filtration (with a 0.2-μm filter).

### HPLC reversed-phase analysis

Of each of the derivative samples described above, 100 mL was injected onto a C18 column (ACE 5 C18 AR 250 × 4.6 mm) on an Alliance HPLC system (Waters) operated at 25 °C. Fluorescence was monitored with a detector at an excitation λ of 390 nm and emission λ filter of 478 nm. Mobile phases used were 10% (v/v) methanol, 0.25% (v/v) acetic acid (solvent A) to 90% (v/v) methanol, 0.25% (v/v) acetic acid (solvent B), at a flow rate of 0.7 mL min^−1^. Upon sample injection, the mobile phase was held at 100% (v/v) solvent A for 7.14 min, then a gradient was applied with sequential composition targets of 90% (v/v) solvent A and 10% (v/v) solvent B for 21.42 min, 80% (v/v) solvent A and 20% (v/v) solvent B for 42.85 min, 20% (v/v) solvent A and 80% (v/v) solvent B for 57.14 min, 100% (v/v) solvent B for 64.28, and 100% (v/v) solvent A for 78.57 min. Data were analyzed by means of the Empower^®^3 software.

### Steady-state kinetics

The steady-state kinetics of AtDHAR2 were measured in a spectrophotometric assay in 0.5-cm and 1.0-cm path-length quartz cuvettes with 100 Bio UV-visible spectrophotometer (Cary) equipped with a temperature-controlled cuvette holder. The AtDHAR2 activity was determined by following the formation of AsA at 265 nm (with a molar extinction coefficient of 7000 M ^1^ cm^−1^) under saturated conditions of DHA (200 μM) with varying concentrations of GSH, ranging from 0.5 mM to 15 mM. Both enzymes and substrates were prepared in assay buffer (300 mM MOPS, pH 7.0, 150 mM NaCl, and 1 mM EDTA). The reaction began with the addition of AtDHAR2-SG at a final concentration of 100 nM to a starting sample mixture of GSH and DHA in a 0.5-mL final volume. All experiments were done in duplicate. One unit of enzyme activity (U) was defined as the amount of enzyme required for the conversion of 1 μmol of substrate into product per minute at 30 °C. The experimental data were fitted to an allosteric sigmoidal equation (Eq. 1), where 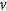 is the steady state velocity, S is the substrate concentration, V_max_ is the maximum rate velocity, *K*_0.5_ is the substrate concentration that gives half the maximal velocity, and h is the Hill coefficient. All the data were analyzed with the GraphPad Prism 6.0 software.


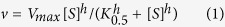


### Fluorescence spectroscopy and stopped-flow analysis

AtDHAR2 contains four tryptophan residues (Trp50, Trp69, Trp171, and Trp207), of which one (Trp207) is located at the H-site and is expected to interact directly with GSH. The overall change in fluorescence of S-glutathionylated AtDHAR2 (prepared as described above) upon rapid mixing with GSH was determined with a SX-20 stopped-flow spectrometer equipped with a fluorescence detector. The final concentration of AtDHAR2-SG was 1 μM in the assay buffer with the GSH concentrations ranging from 0.5 to 17.5 mM. Samples were excited at 295 nm with an emission cut-off of 320 nm. All measurements were done at 30 °C and in triplicate. A single exponential function was used to determine the observable rate constant (*k*_obs_). The *k*_obs_ values were fitted as a function of varied substrate concentration (S) to an equation describing a linear relationship, where *k*_+1_ is the forward and *k*_−1_ the reverse rate constant (Fig. 3A).


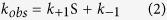


### Crystallization and diffraction data collection

Glutathionylated AtDHAR2 (prepared as described above) at 20 mg mL^−1^ was crystallized by vapor diffusion in a hanging droplet consisting of 1 μL protein, 1 μL precipitant solution (2 M ammonium sulfate, 0.1 M sodium acetate, pH 4.8) at 20 °C. Crystals were cryoprotected by 20% (v/v) glycerol.

X-ray diffraction data were collected on a PILATUS 6 M detector (Dectris) at the Proxima1 beamline of the SOLEIL Synchrotron (Paris). Diffraction data were indexed and integrated in XDS^56^ and the space group of *P*2_1_22_1_ assigned in POINTLESS^57^ based on systematic absences on axial reflections. Scaling and merging of reflections was carried out in AIMLESS^58^ of the CCP4 program suite^59^, with 5% of unique reflections set aside as R-free set.

### Structure determination

Initial phases were determined in Phaser^60^ by molecular replacement with the structure of OsDHAR1 as search model. Initial model building and further iterative structural modifications were carried out in COOT^61^, and maximum-likelihood refinement performed in REFMAC5^62^ and Phenix.refine^63^. Stereochemistry of the model was checked with MolProbity^64^. The Ramachandran plot showed 94.4% of residues to be favored, 5% allowed, and 0.6% generously allowed. Diffraction data collection and refinement statistics are presented in Table S2. The atomic coordinates and structure factors of AtDHAR2 have been deposited in the PDB under the accession code 5LOL.

It is pertinent to highlight the presence of unmodeled electron density at the H-site of AtDHAR2 (Fig. S5). After failed efforts to model components of the purification buffer, mother liquor, or cryoprotectant, an attempt was made to model GSH in this region. Two putative conformations of the H-site GSH were placed, one with the glycinyl terminus oriented inward toward Asp19 and the other in a reverse orientation with the γ-glutamyl directed inward. The modeled GSH gave an acceptable fit to the electron density (Real-space R = 23%) and an average B-factor of 50 Å^2^. However, placement of the H-site GSH was unjustified because of a significant lack of stabilizing protein interactions and relatively unfavorable stereochemistry (bond length/angle root-mean-square Z score of 2.2–4, depending on conformer orientation).

### Structural analysis

Subdomains and hinge axes were identified with the DynDom web server (http://fizz.cmp.uea.ac.uk/dyndom/)^37^. Secondary structure elements were assigned with Stride (http://webclu.bio.wzw.tum.de/stride/)^65^. Normal mode analysis was carried out in the elNémo (http://igs-server.cnrs-mrs.fr/elnemo/index.html)^45^ and ANM 2.0 (http://anm.csb.pitt.edu)^46^ web servers with default parameters. Structural superposition was performed in the Superpose module of CCP4 by means of the Gesamt algorithm. All structural figures were prepared with PyMOL^66^.

## Additional Information

**How to cite this article**: Bodra, N. *et al .Arabidopsis thaliana* dehydroascorbate reductase 2: Conformational flexibility during catalysis. *Sci. Rep.* **7**, 42494; doi: 10.1038/srep42494 (2017).

**Publisher's note:** Springer Nature remains neutral with regard to jurisdictional claims in published maps and institutional affiliations.

## Supplementary Material

Supplementary Information
