# Erratum: *Arabidopsis thaliana* dehydroascorbate reductase 2: Conformational flexibility during catalysis

**DOI:** 10.1038/srep46896

**Published:** 2017-08-24

**Authors:** Nandita Bodra, David Young, Leonardo Astolfi Rosado, Anna Pallo, Khadija Wahni, Frank De Proft, Jingjing Huang, Frank Van Breusegem, Joris Messens

**Keywords:** Enzyme mechanisms, X-ray crystallography

10.1038/srep42494

The original Supplementary Information file published with this Article was incorrect. The correct Supplementary Information now accompanies the Article.

