# Supplementary material for: Arabidopsis thaliana dehydroascorbate reductase 2: Conformational flexibility during catalysis
Source: Sci Rep. 2017 Feb 14;7:42494. doi: 10.1038/srep42494 (PMC5307357; doi:10.1038/srep42494)
Supplement: Supplementary Information [file srep42494-s1.pdf]

SUPPLEMENTARY INFORMATION

Arabidopsis thaliana dehydroascorbate reductase 2: Conformational flexibility during catalysis

Nandita Bodra[1,2,3,4,5,*], David Young[1,2,3,*], Leonardo Rosado[1,2,3], Anna Pallo[1,2,3], Khadija Wahni[1,2,3], Frank De Proft[6], Jingjing Huang[1,2,3], Frank Van Breusegem[4,5] & Joris Messens[1,2,3]

Table S1. Summarized kinetic parameters of plant DHARs and GSTL.

Enzyme	K_m^{GSH} (mM)	K_m^{DHA} (mM)	k_{cat} (s^{-1})	Specific activity (μmol min^{-1} mg^{-1})
**DHAR1[a] (*Oryza sativa*)**	1	0.35	----	350
**DHAR1[b] (*Spinacea oleracea*)**	2.5	0.07	----	370
**DHAR2[c,d] (*Arabidopsis thaliana*)**	3.9 ± 0.4	23 ± 1	7.8 ± 0.4	20 ± 1
**DHAR3A[e] (*Populus trichocarpa*)**	----	0.18 ± 0.02	315 ± 5	----
**GSTL1[f] (*Populus trichocarpa*)**	----	0.09 ± 0.01	1.5 ± 0.027	----

[a] Amako (2006), [b] Hossain (1984), [c] Present work, [d] Waszczak (2014), [e] Lallement (2016), [f] Lallement, (2014)

Table S2. Data collection and refinement statistics for AtDHAR2**.** Statistics for the highest resolution shell are given in parentheses. Ramachandran plot was calculated with MolProbity.

**Data collection**	
Wavelength (Å)	0.9786
Resolution range (Å)	38.73 - 2.3 (2.382 - 2.3)
Space group	$P2_122_1$
Unit cell dimensions (Å)	a = 47.107, b = 67.002, c = 68.026
Total reflections	59565 (2217)
Unique reflections	9622 (733)
Multiplicity	6.2 (3.0)
Completeness (%)	0.96 (0.75)
Mean I/δ(I)	15.01 (1.7)
Wilson B-factor (Å^2)	32.57
R_{merge} (%)	9.996 (67.64)
R_{meas} (%)	10.85 (70.28)
$CC_{1/2}$	0.997 (0.573)
CC*	0.999 (0.854)
**Refinement**	
Reflections used in refinement	9619 (733)
Reflections used for R_{free}	460 (29)
R_{work} (%)	21.18 (26.80)
R_{free} (%)	22.20 (28.57)
CC_{work}	0.938 (0.768)
CC_{free}	0.908 (0.762)
Number of non-hydrogen atoms	1739
Macromolecules	1658
Ligands	31
Protein residues	207
RMS bonds (Å)	0.011
RMS angles (°)	1.34
Ramachandran favored (%)	98
Ramachandran allowed (%)	1.9
Ramachandran outliers (%)	0
Rotamer outliers (%)	53
Clashscore	3.52
Molprobity score	1.14
Average B-factor (Å)	36.25
Macromolecules	36.2
Ligands	41.14
Solvent	34.92

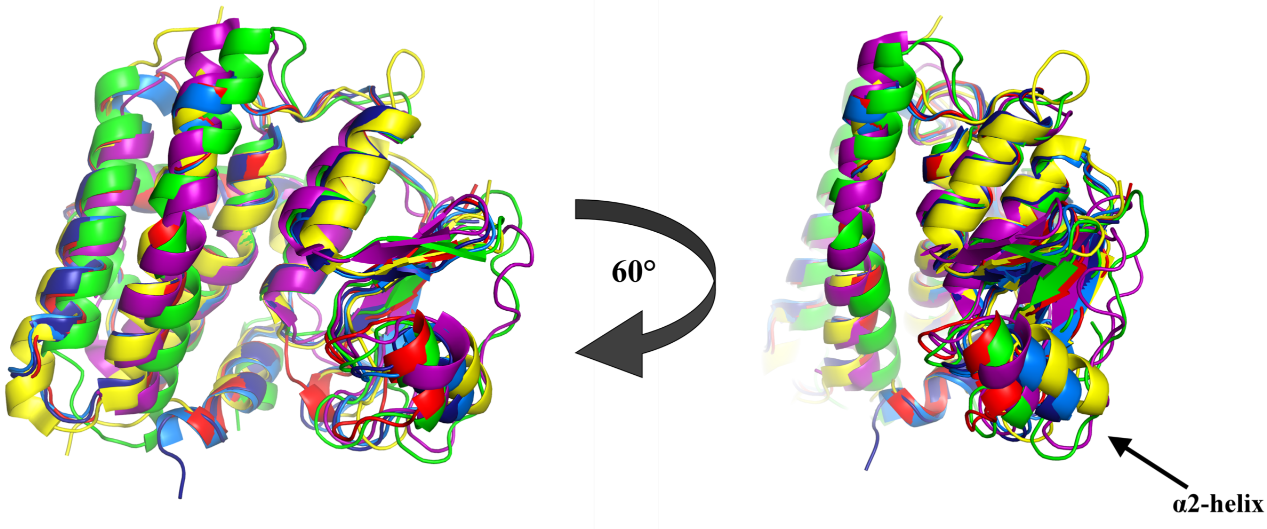

Supplementary Figure S1. The superimposed structures of AtDHAR2 (red), GSTO-2 (green), PtGSTL1 (purple), AtDHAR1 (dark blue), OsDHAR1 (light blue), and CLIC1 (yellow) show a marked conformational variability in the α2-helix region.

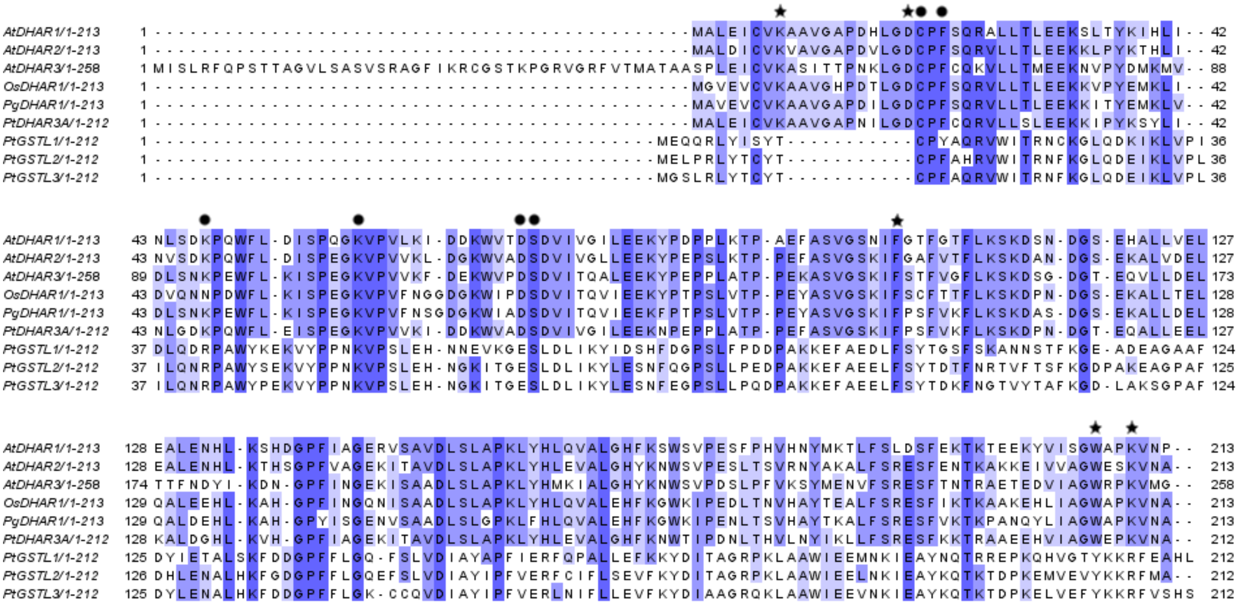

Supplementary Figure S2. Sequence alignment of DHARs from *Arabidopsis* (AtDHAR1-3), *Oryza sativa* (OsDHAR1), *Pennisetum glaucum* (PgDHAR1), and *Populus trichocarpa* GSTL1-3. Residues that engage in side-chain interactions with AsA in the structure of OsDHAR1 (PDB, 5D9W) are indicated by a star. Residues shown in the structure of AtDHAR2 to bind the G-site GSH through side-chain interactions are indicated by a circle.

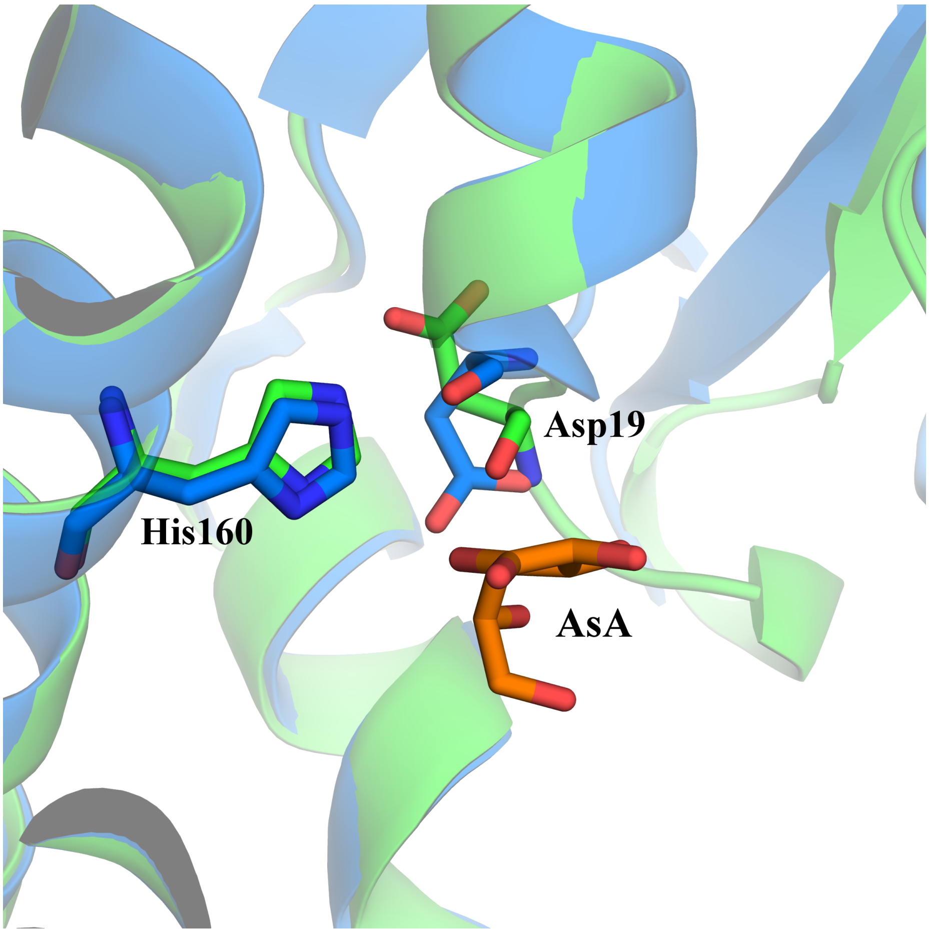

Supplementary Figure S3. Relative conformations of Asp19 in the superimposed structures of AtDHAR2 (green) and AsA-bound OsDHAR1 (blue).

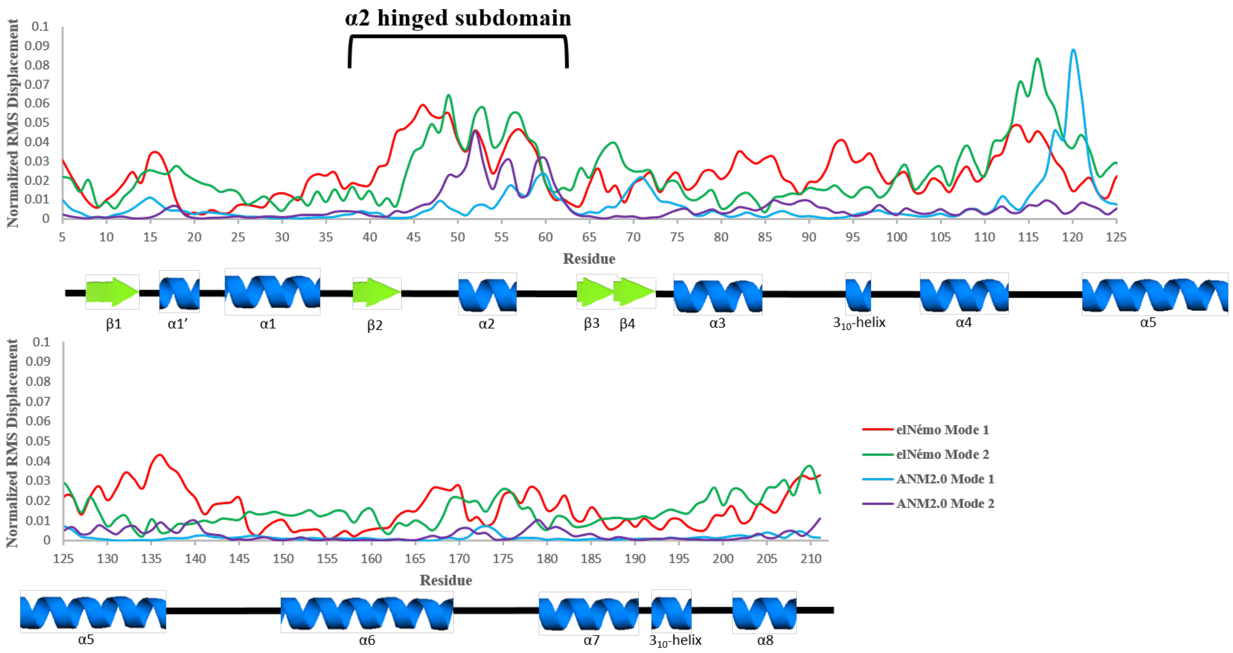

Supplementary Figure S4. Normalized root mean square (RMS) C^{α} displacement across the polypeptide chain of AtDHAR2 in the direction of the two lowest frequency (non-trivial) normal modes calculated by elNémo (red and green) and ANM 2.0 (blue and purple). This provides a measure of structural change at the associated frequency of vibration for the simulation. For clarity, the associated secondary structure elements of AtDHAR2 are presented below the graph.

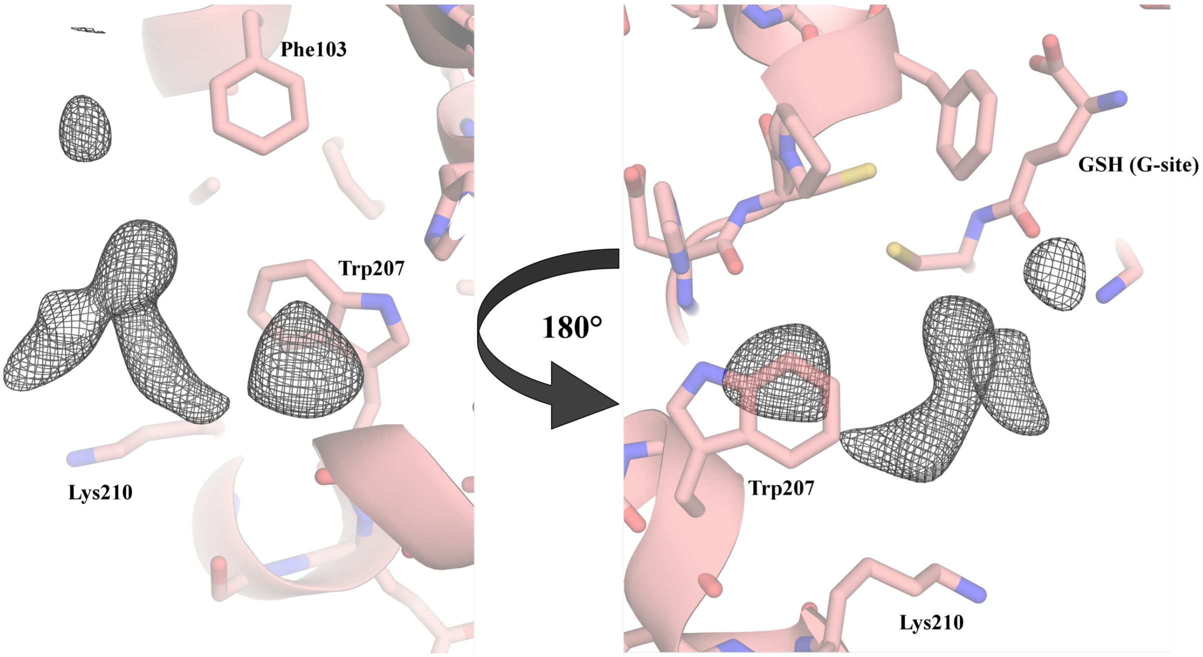

Supplementary Figure S5. Unmodeled density at the H-site of AtDHAR2. The G-site GSH and the H-site residues, Phe103, Lys210, and Trp207 are indicated. Electron density from a mF_O-DF_C difference map contoured at 3 δ is presented as grey isomesh.

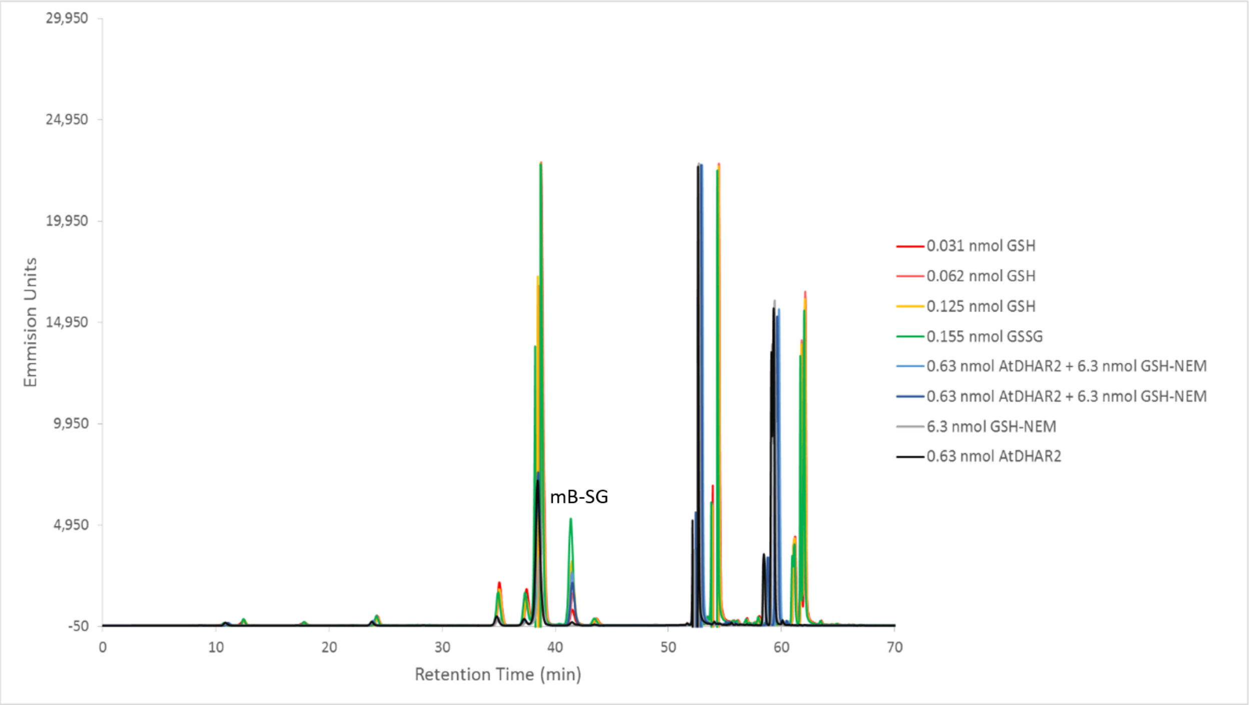

Supplementary Figure S6. Fluorescence trace following the mBBr elution and its derivatives from a reversed-phase C18 column by HPLC. The identity of the emission peak of GSH was assigned according to the retention times of known standards. The fluorescent peak-splitting observable after 50 min is caused by an altered flow rate from 45 min, after elution of the mB-SG derivative.
